# Exploring Parental Hesitancy Toward Childhood COVID-19 Vaccination in the United Arab Emirates

**DOI:** 10.7759/cureus.81697

**Published:** 2025-04-04

**Authors:** Abduljalil Alragheb, Sama Refaei, Dana Hassouna, Maha Altunaiji, Mohamad Khalid Al Aswad, Sumayyah Leila Zaman, Nihar R Dash

**Affiliations:** 1 College of Medicine, University of Sharjah, Sharjah, ARE

**Keywords:** childhood vaccination, covid-19 vaccine, immunization uptake, misinformation, parental hesitancy, public health, uae, vaccine safety

## Abstract

Parental concern regarding COVID-19 vaccination for children remains a significant public health issue. This study examines factors influencing vaccine hesitancy among parents of children aged 5 to 16, focusing on demographics, past vaccination behavior, and trust in information sources. An online cross-sectional survey of 393 parents was conducted. Results showed that children younger than 11 years old were vaccinated against COVID-19 at much lower rates compared to older children, 29.5% of 5-7-year-old children were vaccinated, whereas 97.6% of 14-16-year-old children were vaccinated. Children who had been up-to-date with routine vaccinations were significantly more likely to be vaccinated for COVID-19 (OR = 8.422 among children younger than 11, p < 0.001; OR = 12.113 among children older than 11, p < 0.001). Fathers were more hesitant than mothers (OR = 1.953, p = 0.004), and parents younger than 40 reported greater hesitation than older parents (OR = 2.272, p < 0.001). Interestingly, parents with higher levels of education reported greater hesitation, possibly because they were exposed to misleading scientific data on the internet. Healthcare practitioners were trusted by 76.8% of parents who sought vaccine information from their child's doctor, compared to 20.3% who looked to social media. Concerns about vaccine safety, including the newness of the vaccine and side effects, were highly prevalent. The implications of these findings suggest a need for public health campaigns to clear up misinformation, emphasize trusted clinical sources, and reach out to groups showing hesitation, particularly younger and educated parents. Cultural beliefs and psychological constructs, such as risk perception and trust in institutions, shape vaccine hesitancy, making it essential to identify key demographic and psychological factors to improve vaccine confidence and achieve greater uptake among United Arab Emirates (UAE) parents.

## Introduction

After the US FDA authorized the Pfizer-BioNTech and Moderna COVID-19 (SARS-CoV-2) vaccines for emergency use in children aged between 6 months and 4 years, some trends of hesitancy among different segments of society could be noted. Parental resistance and doubtfulness regarding the effectiveness of the available vaccines have contributed greatly to this hesitancy [1]. The authorization of vaccine administration in children had the main objective of shielding them from the possible complications of infection. Furthermore, it also aims to provide adequate protection to family members who might encounter an infected child. The administration of the vaccine itself can further reduce the severity and criticalness of symptoms that may arise in family members [2]. High vaccination rates and implementation in children and adults can be a successful factor, contributing to the mitigation of infection in schools and workplaces, where mask removal is very common [3]. The burden of infection and efficient attainment of herd immunity can be significantly propagated by the implementation of child vaccination [2].

Vaccine hesitancy is a multidimensional issue affected by several key factors, such as pre-existing beliefs about vaccines, past experiences with them, the marital status of the subjects, and their socioeconomic status [4,5]. Based on events that occurred during the COVID-19 pandemic, both UNICEF and the WHO issued a warning, highlighting the concerning drop in the number of child vaccinations. This decline poses a significant threat as it increases the risk of preventable diseases, and the issued warning emphasizes the benefit of prioritizing routine vaccinations [6].

The primary aim of this study was to comprehensively assess parental hesitancy toward administering the COVID-19 vaccine to children aged 5-16 in the United Arab Emirates (UAE). Another objective was to examine the demographic, personal, and environmental factors influencing parental decision-making, including concerns about side effects, vaccine effectiveness, and levels of trust in healthcare authorities and information sources. By analyzing these factors, the study sought to identify the key drivers of vaccine hesitancy among UAE parents. These objectives have important public health implications in the UAE, offering evidence to guide national strategies, shape awareness campaigns, and support vaccine uptake. Ultimately, this can contribute to better protection of children, higher immunization rates, and improved population-level immunity against future outbreaks.

## Materials and methods

Study participant and sampling technique

This study used a cross-sectional design to assess UAE parents' hesitancy towards having their children vaccinated against COVID-19. The study population included parents in the UAE with children aged 5 to 16 years who have access to the internet. Data were collected using a volunteer sampling method. It is essential to acknowledge that using a volunteer sampling method may introduce selection bias, as parents with strong opinions about COVID-19 vaccination may be more likely to participate. Parents with children aged between 5 and 16 years who can speak either Arabic or English were eligible to participate. Children with clear contraindications for the vaccine were excluded from the study. The required sample size was 315, calculated using the formula n = 4p(1-p)/SE², where p was 0.27 based on a similar study in Saudi Arabia [7], and the margin of error (SE) was 5%. An additional 10% was added for non-responses, bringing the final required sample size to 347.

Data collection tools 

Google Forms was used to conduct a self-administered online questionnaire consisting of 22 questions, collected at a single point in time over 4 months between February 2022 and May 2022. We adapted questions from a previous study and ensured cultural appropriateness through expert review, removing potentially sensitive questions [8]. The questionnaire was originally in English and translated into Arabic by native Arabic-speaking researchers. The translation was then reviewed by a group of bilingual individuals to ensure clarity, accuracy, and cultural relevance. Adjustments were made where necessary to maintain the intended meaning of the original questions. We distributed the questionnaire over several mainstream social media platforms such as WhatsApp, Telegram, and Twitter. The questionnaire consisted of three sections. The first section includes two domains: the first domain focuses on parent demographic characteristics, such as gender, age group, nationality, educational level, employment in the medical field, number of children, and family income. The second domain targets child-related data, including the number of children aged between 5 and 16 years, their age group, gender, vaccination status, COVID-19 vaccine status, and reasons for not vaccinating their children against COVID-19. The second section comprises a ten-statement Likert-type scale aimed at measuring parent perceptions about COVID-19 vaccines for children. The last section contains seven sources of information about the COVID-19 vaccine, also presented in a Likert-type scale, focusing on measuring parents’ level of trust. The parents' perceptions about vaccines were scored as 1 = strongly agree, 2 = somewhat agree, 3 = unsure/I don’t know, 4 = somewhat disagree, and 5 = strongly disagree. The scale measuring parents’ level of trust in sources of information was scored as 1 = trust completely, 2 = trust mostly, 3 = trust somewhat, 4 = do not trust, and 5 = not applicable.

Statistical analysis

Data analysis was performed using SPSS Statistics version 24. Frequency tables were used to describe categorical variables, while means and SDs were used to summarize continuous variables. Chi-square tests were employed to examine the relationship between parental hesitancy and various sociodemographic factors (gender, age, educational level, nationality, occupation, income) as well as child-related factors, including age and vaccination status. Odds ratios were calculated to assess vaccine hesitancy across different groups. Statistical significance was set at p < 0.05. While chi-square tests identify associations, they do not account for potential confounding factors. Future studies should consider using multivariate analyses, such as logistic regression, to better understand the independent effects of each variable on vaccine hesitancy.

Ethical considerations 

The study received ethical approval from the Research Ethics Committee at the University of Sharjah (Reference number: REC-22-02-17-S). Informed consent was obtained from all participants. Participation in the study was voluntary, and completing the questionnaire indicated the participant’s consent to take part. All data collected remained anonymous and confidential, with the assurance that it would only be used for research purposes.

## Results

The study included 393 parents residing in the UAE, with children aged 5 to 16 years. The majority of respondents were female (70%), and the largest proportion of parents (39.4%) were between 41 and 50 years old. Arab parents represented the majority of the sample (63.4%), and nearly half of the parents (47.8%) held a bachelor’s degree. Most parents (77.1%) were not employed in healthcare-related occupations, and 35.1% reported a monthly income of more than 25,000 AED. The age distribution of the children in the study revealed that the largest group consisted of children aged 14-16 years (52.7%), followed by those aged 11-13 years (50.6%), 8-10 years (42.0%), and 5-7 years (37.9%) (Table 1).

**Table 1:** Parental sociodemographic information.

Characteristics	Frequency	%
Gender		
Male	118	30%
Female	275	70%
Parent Age Group		
20-30	64	16.30%
31-40	127	32.30%
41-50	155	39.40%
Above 50	47	12.00%
Nationality		
UAE Nationals	105	26.90%
Other Arab Nationals	248	63.40%
Non-Arab Nationals	38	9.70%
Educational Level		
High School or Less	79	20.10%
Diploma	38	9.70%
Bachelor’s Degree	188	47.80%
Postgraduate Degree	88	22.40%
Employed in Healthcare		
Yes	90	22.90%
No	303	77.10%
Monthly Income		
Less than 5,000 AED	21	5.30%
5,000 AED - 9,999 AED	52	13.20%
10,000 AED - 14,999 AED	72	18.30%
15,000 AED - 19,999 AED	44	11.20%
20,000 AED - 24,999 AED	66	16.80%
More than 25,000 AED	138	35.10%
Currency Conversion	*1$ = 3.67 AED	

The highest rate of up-to-date vaccination schedules was observed in the 14-16-year-old age group (92.8%), followed by the 11-13-year-old age group (91.5%), the 5-7-year-old age group (89.3%), and the 8-10-year-old age group (87.9%). These results indicate that most parents in the UAE adhere to routine childhood vaccination schedules, regardless of their hesitancy toward the COVID-19 vaccine. However, parental hesitancy toward COVID-19 vaccination decreased as the age of the child increased.

The vaccination rates were 29.5% for children aged 5-7 years, 36.4% for those aged 8-10 years, 55.8% for those aged 11-13 years, and 97.6% for those aged 14-16 years. This trend suggests that hesitancy is higher for younger children, likely due to concerns about vaccine safety and availability for younger age groups.

Children who were up-to-date on their routine childhood vaccinations were significantly more likely to receive the COVID-19 vaccine. Chi-square tests revealed that for children below the age of 11, those who had completed their childhood vaccinations were more than eight times more likely to be vaccinated against COVID-19 (odds ratio = 8.422, p = 0.001). On the other hand, children above the age of 11, those who were up-to-date on their childhood vaccinations were more than twelve times more likely to receive the COVID-19 vaccine (odds ratio = 12.113, p < 0.001). These findings highlight the strong association between adherence to routine vaccinations and acceptance of the COVID-19 vaccine.

Compared to male parents (61.9%), female parents were significantly more inclined to vaccinate their children (76.0%). Chi-square analysis revealed that males were nearly twice as likely to be hesitant about vaccinating their children compared to females (odds ratio = 1.953, p = 0.004). Parents' age played a significant role in vaccine hesitancy. Those over 40 were much more likely to vaccinate their children, with a rate of 79.7%, compared to just 63.4% among younger parents under 40. A chi-square test showed that parents above 40 were more than twice as likely to choose vaccination (odds ratio = 2.272, p = 0.001). In contrast, other demographic factors such as education level, occupation, and income did not show a strong connection to vaccine hesitancy. For instance, vaccination rates were nearly the same among parents with a postgraduate degree (72.6%) and those with a high school diploma or lower (70.1%). Similarly, there were no notable differences based on income or whether a parent worked in healthcare.

**Figure 1:**
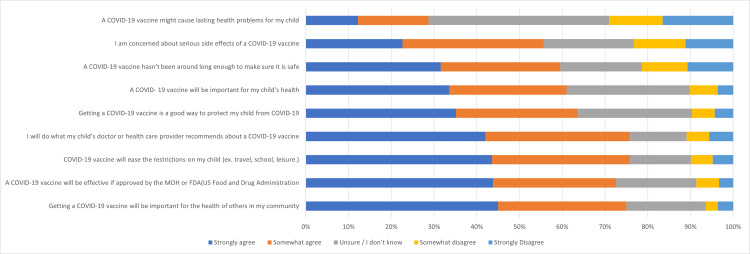
Parental hesitancy and perceptions toward COVID-19 vaccination for their children.

A high proportion of parents agreed that the vaccine would be beneficial, effective, and important for their child’s health and the health of others. Specifically, 61.1% of parents strongly or somewhat agreed that a COVID-19 vaccine would be important for their child’s health, and 72.6% agreed that it would be effective if approved by the MOH or FDA. However, many parents had significant concerns about vaccine safety. About 28.7% believed that a COVID-19 vaccine could potentially cause long-term health problems for their child, and 55.7% were worried about serious side effects. In addition, 59.6% felt that the vaccine had not been available long enough to be fully certain of its safety. This highlights how the vaccine's relatively recent introduction contributed to widespread hesitancy (Figure 1).

**Figure 2:**
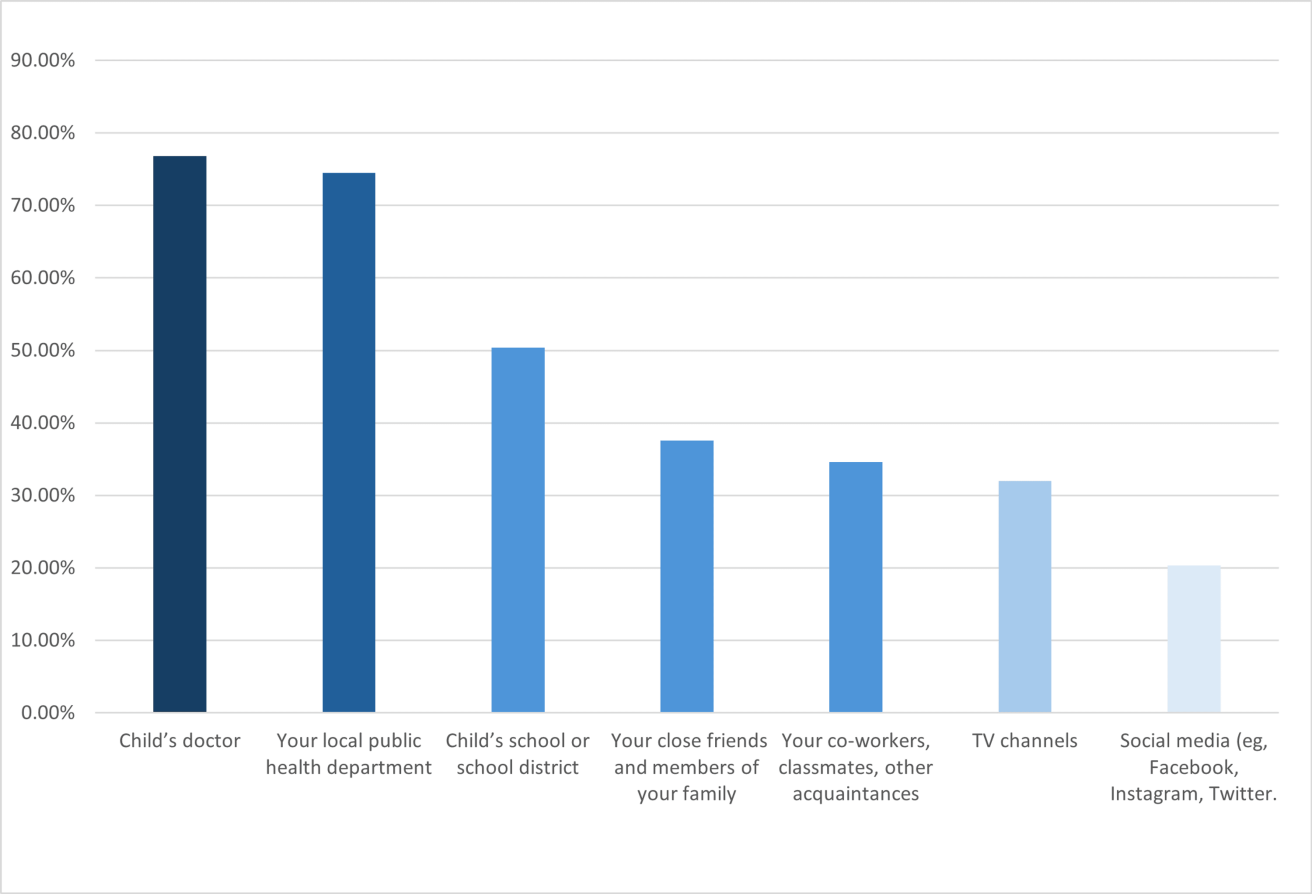
Parents' trust in various sources of information about the COVID-19 vaccine.

The most trusted sources about vaccines were their child’s doctor (76.8% completely or mostly trusted) and their local public health department (74.5% completely or mostly trusted). When it came to trust in media, only 20.3% of parents reported having complete or high confidence in social media platforms, while 39.7% said they did not trust them at all. A similar pattern was observed with TV channels, which were trusted by 32.0% of parents, whereas 28.2% expressed no trust in them. These findings emphasize the importance of healthcare professionals and official health organizations as the most trusted sources of vaccine-related information (Figure 2).

## Discussion

This research examines the various reasons that led parents in the UAE to be hesitant about vaccinating their children against COVID-19. The results show how age, gender, education, and prior vaccinations significantly impact their decision regarding the COVID-19 vaccine. This discussion seeks to understand the implications of vaccine hesitancy and its impact on public health by presenting the quantitative results derived from our bivariate analysis.

According to our research, mothers were significantly more likely than fathers to get their children vaccinated (OR = 1.953, p = 0.004). This is supported by studies showing that mothers generally have more favorable attitudes toward vaccinations and greater willingness to vaccinate their children compared to fathers [9]. However, some researchers present contrasting findings, suggesting that fathers may be more likely to vaccinate their children. Additionally, parental vaccination decisions may be influenced by ecological and individual factors beyond gender alone [10, 11]. In order to provide focused and customized public health interventions, evidence-based counseling and communication campaigns specifically addressing these concerns are required to properly educate parents and prevent the spread of misleading information.

The choice to vaccinate children was affected by parental age. Parents younger than 40 were much less willing than those over 40 to vaccinate their children (OR = 2.272, p < 0.001). This is consistent with previous studies stating that older parents tend to have less hesitancy towards COVID-19 vaccines, possibly because of their greater exposure to immunization programs, and it could be correlated with them having older children. In contrast, younger parents tend to have misinformation, particularly those spread through social networks [11]. Hence, targeted campaigns aided by education are needed for this demographic. The concerns of younger parents can be addressed through community workshops, webinars, and social media campaigns to increase vaccination rates [1].

This study further revealed that parents who had only completed high school or are less educated were more inclined to vaccinate their children than those with higher education (OR = 2.571, p = 0.004). We speculate that those educated to a higher level may be more susceptible to vaccine misinformation, especially from internet sources that misuse scientific evidence, or due to their concern about serious vaccine side effects and the novelty of the vaccine. More studies are needed to investigate this correlation. However, other studies have found the opposite, in which a higher educational level was associated with a higher willingness to vaccinate their children [11, 12].

Remarkably, vaccine hesitation was not statistically significantly impacted by nationality, occupation, or income. Working in the healthcare industry did not correlate with greater vaccination rates (p = 0.705), nor did nationality significantly influence vaccination decisions (p = 0.224). This study implies that when it comes to vaccine decision-making, cultural background and professional knowledge may be subordinated to personal opinions and exposure to false information. Likewise, there was no significant correlation between parental income and reluctance (p = 0.326), suggesting that socioeconomic position may not have as much of an impact on vaccine acceptance [13].

Furthermore, there was a relationship between the uptake of the COVID-19 vaccine and past childhood immunization history. At ORs of 8.422 for children under 11 and 12.113 for those over 11, children who had received their regular childhood vaccines had a significantly higher chance of being immunized against COVID-19 (p-values 0.001 and 0.001, respectively). The significance of establishing and preserving confidence in children's vaccination programs is highlighted by this study. Public health campaigns should incorporate COVID-19 vaccine information into regular immunization conversations and highlight the proven effectiveness of vaccination in preventing illnesses [13, 14].

Moreover, compared to younger children (36.7%), older children (74.3% of those over 11) had higher percentages of both childhood and COVID-19 immunizations [15, 16]. This suggests that, either as a consequence of increased parental knowledge or exposure to school-based vaccination programs, parents are more likely to vaccinate their children as they age. Vaccine decisions were found to be significantly influenced by trust in medical practitioners. While 74.5% of parents trusted their local public health authorities, the majority of parents (76.8%) trusted their child's physician. Conversely, there was significantly less trust in acquaintances and social media [17]. This emphasizes the importance of healthcare professionals in influencing vaccine attitudes [18].

The findings from our study align with the more general global patterns of vaccine hesitancy, according to a comparison with data from other countries. Middle Eastern nations, such as Kuwait and Jordan, had some of the lowest acceptance rates worldwide (23.6% and 28.4%, respectively), as noted in a systematic review of COVID-19 vaccine acceptance rates across 33 countries [16]. Parental vaccine hesitancy is also evident in the UAE, particularly among younger and better-educated parents. This is consistent with the regional trend of hesitancy driven by misinformation and mistrust. The Middle East demonstrated comparatively lower vaccine acceptance rates, while countries in East and Southeast Asia reported levels exceeding 90%. These disparities underscore the critical influence of sociocultural and informational contexts, highlighting the need for culturally tailored public health communication strategies to enhance vaccine confidence [16].

The strengths of this study include addressing a timely and critical public health issue, employing rigorous statistical methods (chi-square tests, ORs, and p-values), and using a diverse participant pool with a reasonable sample size of 393 parents. Additionally, the use of a bilingual and culturally appropriate questionnaire ensured clarity and relevance for participants, reducing the likelihood of misunderstanding. Exploring both general childhood vaccination practices and COVID-19 vaccine attitudes provided a broader understanding of parental decision-making. These strengths contribute useful evidence to support future health education and vaccination campaigns in the region.

While this study provides valuable insights, several limitations must be considered. Self-reported data might be subject to bias due to recollection inaccuracies or social desirability. The higher proportion of female respondents compared to males may limit the biological significance and generalizability of gender-related findings, making it harder to detect significant differences between genders. Furthermore, most participants were Arab, limiting generalizability to other ethnic groups within the UAE; future research should aim for broader representation [19]. Another limitation is that the specific content and sources of misinformation, such as social media, peer influence, or misinterpreted scientific content, were not explored in detail. Further research is warranted to explore the underlying reasons for the observed positive association between higher educational levels and vaccine hesitancy, particularly given that our findings contrast with those of previous studies [11, 12].

In conclusion, the findings of the study suggest several solutions for developing effective public health interventions: Since misinformation disproportionately impacts female and younger parents, programs should first concentrate on resolving vaccination reluctance among these groups through tailored communication tactics [17]. Second, healthcare professionals should play a bigger role in vaccination advocacy, as they are some of the most trusted sources of information about vaccines [17,18]. Last but not least, governments and health organizations must offer training to ensure that physicians, nurses, and other healthcare providers have the skills required to engage with parents who are apprehensive about vaccinations. Strategies for debunking misinformation should be developed. Collaborating with social media platforms to detect deceptive content and highlight reliable sources of vaccine information can reduce the spread of false information. This study indicates that factors like gender, age, education level, and past vaccination history greatly impact parents' reluctance to vaccinate their children against COVID-19 in the UAE [15,16]. Public health programs ought to focus on younger, more educated female parents, as these demographics show greater reluctance [15].

Future research should delve deeper into the social, cultural, and psychological reasons behind vaccine hesitancy by using interviews and focus groups to capture parents' real concerns and experiences [18]. Expanding studies to include a more diverse range of parents from different backgrounds in the UAE would also provide a clearer picture of vaccine attitudes across various communities [15]. Additionally, exploring the long-term effects of misinformation and evaluating the success of public health campaigns can help improve future strategies. In the end, tackling vaccine hesitancy requires more than just facts, it encompasses building trust, addressing concerns with empathy, and keeping communities well-informed [16,17].

## Conclusions

While recorded adherence to the general vaccination schedule for children was noted, COVID-19 vaccine hesitancy persists. Younger and male parents were observed to be more hesitant, whereas older parents were more accepting. Paradoxically, higher education was associated with lower vaccination rates, possibly influenced by the misuse of scientific evidence online or concerns over serious vaccine side effects and the novelty of the vaccine. However, this finding contrasts with studies where higher education correlated with increased vaccine acceptance, highlighting the need for further research on this topic. The observed gender differences should be interpreted cautiously, as the higher proportion of female respondents limits the biological significance and generalizability of these findings. To address vaccine hesitancy effectively, public health initiatives should employ tailored strategies for specific groups. For highly educated individuals, targeted campaigns could focus on delivering transparent, evidence-based messages via trusted platforms such as professional healthcare organizations or academic webinars. For hesitant parents, healthcare providers should initiate personalized discussions during routine health visits, emphasizing vaccine safety and dispelling misinformation. Social media campaigns should focus on debunking common myths using culturally relevant messaging and endorsements from local influencers or trusted community leaders. Integrating vaccine education into school and workplace programs could also ensure widespread awareness. Future studies should investigate the content and sources of vaccine misinformation, exploring how they specifically influence vaccine hesitancy among highly educated individuals.

## Figures and Tables

**Figure 3:**
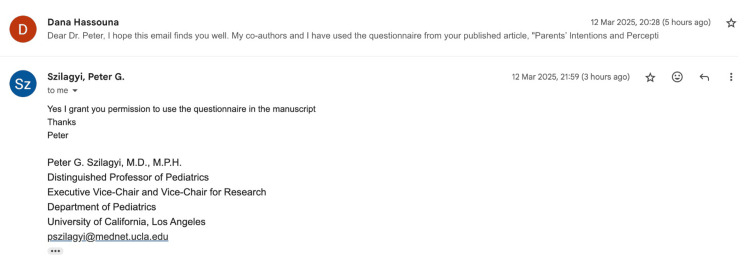
Email granting permission for questionnaire use.

## Appendices

Research questionnaire attribution and permission 

This study utilized a modified version of the questionnaire from the following article: Szilagyi PG, Shah MD, Delgado JR, et al.: Parents’ intentions and perceptions about COVID-19 vaccination for their children: results from a national survey. Pediatrics. 2021, 148: e2021051452. 10.1542/peds.2021-052335 [8].

As the publisher does not issue formal permission numbers, authorization was obtained directly from the original author, Dr. Peter G. Szilagyi, via email correspondence. A copy of the approval email is included below as proof of authorization.

Sociodemographic characteristics of study sample

**Table 2:** Parent characteristics.

Question	Options
1. What is your gender?	Male / Female
2. Specify the age group you belong to:	20-30 / 31-40 / 41-50 / Above 50
3. What is your nationality?	____________________________
4. What is your highest educational level?	Did not complete high school / High school / Diploma / Bachelor’s degree / Postgraduate degree (master, PhD, etc.)
5. Do you work in the medical field?	Yes / No
6. What is your total family income per month?	Less than 5,000 AED / 5,000 AED – 9,999 AED / 10,000 AED – 14,999 AED / 15,000 AED – 19,999 AED / 20,000 AED – 24,999 AED / More than 25,000 AED

Child-related data

The following questions pertain to your children who are between the ages of 5 and 16 years old.

**Table 3:** Number of children aged 5-16 years.

Question	Response Options
7. How many children do you have between 5 and 16 years?	1, 2, 3, 4, more than 5

**Table 4:** Age and gender of children.

Child Number	Age Group	Gender
1st Child	5-7 / 8-10 / 11-13 / 14-16	Male / Female
2nd Child	5-7 / 8-10 / 11-13 / 14-16	Male / Female
3rd Child	5-7 / 8-10 / 11-13 / 14-16	Male / Female
4th Child	5-7 / 8-10 / 11-13 / 14-16	Male / Female
5th Child or More	Please enter the ages of your children: ______	Male / Female

**Table 5:** Vaccination status.

Question	Response Options
My child’s vaccination is up to date	Yes / No
Has your child received the COVID-19 vaccine?	Yes / No

**Table 6:** Reasons for not vaccinating children against COVID-19.

Question	Response Options
What are the reasons for not vaccinating your children against COVID-19?	Open-ended response

Perceptions about COVID-19 vaccines for children

**Table 7:** Perceptions about COVID-19 vaccines for children.

Statement	1) Strongly Agree	2)Somewhat agree	3) Unsure / I don’t know	4) Somewhat disagree	5) Strongly disagree
A COVID-19 vaccine might cause lasting health problems for my child.					
I am concerned about serious side effects of a COVID-19 vaccine.					
A COVID-19 vaccine hasn’t been around long enough to make sure it is safe.					
I will do what my child’s doctor or health care provider recommends about a COVID-19 vaccine.					
Getting a COVID-19 vaccine will be important for the health of others in my community.					
A COVID-19 vaccine will be effective if approved by the MOH or FDA(US Food and Drug Administration).					
Getting a COVID-19 vaccine is a good way to protect my child from COVID-19. **					
A COVID- 19 vaccine will be important for my child’s health.					
COVID-19 vaccine will ease the restrictions on my child (ex. travel, school, leisure).					

Parents’ level of trust in sources of information about COVID-19 vaccine

**Table 8:** Parents’ level of trust in sources of information about the COVID-19 vaccine.

Sources of information about COVID-19 vaccine	1) Trust Completely	2) Trust Mostly	3) Trust Somewhat	4) Do Not Trust	5) Not Applicable
Child’s doctor					
Child’s school or school district					
Your local public health department					
Your close friends and members of your family					
Your co-workers, classmates, other acquaintances					
Social media (eg, Facebook, Instagram, Twitter)					
TV channels					
